# A Pharmacoeconomic Outlook of the Biological Drugs Marketed in India: A Cost Variance Analysis

**DOI:** 10.7759/cureus.33943

**Published:** 2023-01-18

**Authors:** Viraj A Shinde, Ramanand Patil, Prasan Bhandari, Prachitee Borkar, Shraddha Yadav

**Affiliations:** 1 Department of Pharmacology, Symbiosis Medical College for Women (SMCW) , Symbiosis International (Deemed University) (SIU), Pune, IND

**Keywords:** cost-benefit analysis, drug prices control order, cims, biosimilars, biologics, autoimmune diseases

## Abstract

Introduction

Biologic drugs are used to treat various illnesses like autoimmune diseases, cancers, hormonal irregularities, anemia, etc., and to prevent various diseases as vaccines. Though various biologic drugs are already available, they are still not within reach of the common man due to financial constraints. Through many search engines, studies evaluating the cost variation of different brands of biologics were investigated; however, only a few studies that address this problem were found. Hence, this study was planned with the objective of addressing the cost variation of various brands of biologic medicines available in the Indian market.

Methods

The website for the Current Index of Medical Specialties (CIMS) for India’s location was used to obtain the prices of the different brands of biologic medicines in Indian National Rupee (INR) currency, which different manufacturers market with identical forms in strength and dosage. The percentage cost variation and cost ratio were calculated with the help of the minimum and maximum prices of various brands of biologic drugs.

Results

The prices of biologics belonging to six different classes that are available in 23 formulations were analyzed. The highest cost variability was shown by pegfilgrastim 6 mg at 1,022.92%, and the minimum-cost variation was shown by darbepoetin alfa 200 mcg at 13.07%.

Conclusion

Our research found a vast variance in the costs of various brands of biologic medicines in India. The government should address this cost variation problem by developing various policies, such as breaking the monopoly of manufacturers, providing tax incentives to nonprofit generic medicine manufacturers, and incorporating more biologic drugs under the protection of the Drugs Prices Control Order (DPCO).

## Introduction

Biologics are defined as a heterogeneous group of proteins, drugs, and or derivatives that regulate the inflammatory response, modify the immune system, or support tumor-specific defenses [1]. “Biologicals” or “recombinant therapeutics” are other names for biologics [1]. Biologicals are all drugs that have been derived from different biological sources, e.g., humans, animals, or microorganisms [2]. They include growth factors, pro- and anti-angiogenic factors, monoclonal antibodies, fusion and recombinant proteins, and expression vectors that generate proteins in situ [1]. Biosimilars (similar biologics) are biological products or medicines manufactured by genetic engineering technology and are considered to be comparable in various aspects to reference biologics regarding safety, efficacy, and quality. These reference biologics have been granted permission by the Drug Controller General of India (DCGI) for safe usage [3]. The contents of a biosimilar medicine are the same as that of reference biologics. They are both used in identical dosage forms in managing the same diseases [3]. The last 30 years have seen the development of various biologic drugs [3]. The production of follow-on biologics in the USA or biosimilars in the UK was initiated as a result of the cessation of the patent protection of several biological drugs [3]. Furthermore, biologics have targeted action that can help deliver personalized therapy to every patient [4]. They are the most recently developed therapies used to manage various disease conditions. Biologicals and biosimilar drugs have changed the treatment and management of various diseases like cancers, rheumatoid arthritis, psoriasis, psoriatic arthritis, ulcerative colitis, Crohn’s disease, multiple sclerosis, ankylosing spondylitis, systemic lupus erythematosus, and type 1 diabetes mellitus [4-7].

On the other hand, cancer cells selectively express specific target molecules that are involved in their progression and survival. These can be repressed by biological drugs [4]. The selective action of biologics helps in preserving healthy tissue. This selective action also decreases the adverse effects caused by the general cytotoxicity of conventional anticancer therapy [4]. Biologics very effectively manage many diseases; however, their use in treating a disease is highly restricted due to their high costs [8]. The advent of biosimilars has resulted in the decreasing cost of the treatment [8]. Being an underdeveloped country, the cost of drugs in India is a significant issue. The majority of physicians in India prescribe using brand names [9]. Consequently, this ultimately results in the prescription of drugs with high costs. This often results in the patient’s noncompliance with the treatment [10,11], leading to increased morbidity. This also places strain on the physician-patient relationship [12]. In emergent nations like India, where health insurance is at a premium, the full cost of treatment is often borne by the patient [13]. Pharmacoeconomics plays a significant role in the management of a patient’s disease. The cost of medicines not only has a significant impact on the treatment compliance of an illness, but it also plays an important role in rational drug use [9]. Currently, there are few studies on the analysis of cost variation of different brands of biological drugs in India that are published in the literature [14,15]. Hence, this study has been undertaken to analyze the cost differences of various brands of biological drugs so that less costly but similarly effective brands can be prescribed whenever possible.

## Materials and methods

The study commenced after it was approved by an independent ethics committee, Symbiosis International (Deemed University), Pune, India. Its approval number is SIU/IEC/217. The cost of various biological drugs was acquired from an online resource, the Current Index of Medical Specialties (CIMS) website. Its location was set in India during January-March 2022. These biologic drugs are frequently prescribed and are available in the Indian pharmaceutical market based on the CIMS database. The analysis excluded the cost of the medicines and formulations that were not listed in the above source. The cost of drug formulation in the same strength with the same route was compared. Biologic drugs were segregated and analyzed into classes based on the Anatomic Therapeutic Classification (ATC) system.

The minimum and maximum prices in rupees (INR) for specific biological medications that were marketed by several pharmaceutical businesses in identical strength were also recorded. The next step was calculating the cost ratio from the costliest to cheapest brands of the same medicine in the same strength. This will show how much more expensive the costliest brand in each category is compared to the least expensive. In addition, the percentage cost variation was calculated using the formula: % cost variation = (maximum cost - minimum cost)/minimum cost * 100 [14]. Drug formulations that were manufactured by only one company were excluded.

Statistical analysis

Microsoft Excel® 2016 was used to examine the data from the CIMS website. Absolute numbers and percentages were used to express the study’s findings. The cost ratio and cost variation percentage were determined. Correlation analysis was used to establish the relationship between the number of brands and percentage cost variation.

## Results

Various biological drugs were searched in CIMS for their prices; however, only 12 different biologics could be found with pricing. We analyzed the cost of the 12 different biologics from six distinct classes available in 23 different formulations. Somatropin injection 4 IU showed a variability of 182.25% (Table 1).

**Table 1:** The cost variation among the class of somatropin and somatropin agonists. mg: Milligram, IU: International unit; INR: Indian national rupees; US$: United States Dollar; 1.00 US$ = 82.72 INR. Source: reference 16.

Drug (ATC code)	Dosage form	Dose	Number of brands	Minimum price INR (US$)	Maximum price INR (US$)	Cost ratio	Percentage cost variation
Somatropin (H01AC01)	Injection	4 IU	3	1,550 (18.74)	4,375 (52.89)	2.82	182.25

Table 2 shows the cost variation in the “other anti-anemic drugs class.” In this class, the maximum cost variation was shown by the drug darbepoetin alfa in the case of 40 mcg, which was at 54.28%. The minimum cost variation was also shown by darbepoetin alfa formulation 200 mcg, which was at 13.07%.

**Table 2:** The cost variation among the drugs belonging to class of “other antianemic drugs.” mg: Milligram, IU: International unit; INR: Indian national rupees; US$: United States Dollar; 1.00 US$ = 82.72 INR. Source: reference 16.

Drug (ATC code)	Dosage form	Dose	Number of brands	Minimum price INR (US$)	Maximum price INR (US$)	Cost ratio	Percentage cost variation
Darbepoetin alfa (B03XA02)	Injection	25 mcg	15	1,500 (18.13)	2,025 (24.48)	1.35	35
	Injection	40 mcg	18	1,914 (23.14)	2,953 (35.69)	1.54	54.28
	Injection	60 mcg	5	3,500 (42.31)	4,900 (59.24)	1.4	40
	Injection	100 mcg	2	3,125 (37.78)	4,722 (57.08)	1.51	51
	Injection	200 mcg	2	9,285.71 (112.25)	10,500 (126.93)	1.13	13.07
Epoetin alfa (B03XA01)	Injection	4,000 IU	2	960 (11.61)	1,332 (16.10)	1.38	38.75
	Injection	10,000 IU	2	2,350 (28.40)	2,660 (32.16)	1.13	13.2

In the class of “colony-stimulating factors,” 6 mg pegfilgrastim showed a maximum cost variation of 1,022.92%, and the minimum cost variation in it was shown by 300 mcg filgrastim injection, which was 141.99% (Table 3).

**Table 3:** The cost variation among the drugs belonging to the class of “colony-stimulating factors.” mg: Milligram, IU: International unit; INR: Indian national rupees; US$: United States Dollar; 1.00 US$ = 82.72 INR. Source: reference 16.

Drug (ATC code)	Dosage form	Dose	Number of brands	Minimum price INR (US$)	Maximum price INR (US$)	Cost ratio	Percentage cost variation
Pegfilgrastim (L03AA13)	Injection	6 mg	14	3,010 (36.39)	33,800 (408.61)	11.22	1,022.92
Filgrastim (L03AA02)	Injection	300 mcg	19	1,162.50 (14.05)	2,812.50 (34)	2.41	141.99

In the class of “monoclonal antibodies,” the maximum cost variation was shown by 440 mg of trastuzumab, which was 275.09%; the minimum was also shown by 150 mg of trastuzumab, which was 17.32% (Table 4).

**Table 4:** The cost variation among the drugs belonging to the class of “monoclonal antibodies.” mg: Milligram, IU: International unit; INR: Indian national rupees; US$: United States Dollar; 1.00 US$ = 82.72 INR. Source: reference 16.

Drug (ATC code)	Dosage form	Dose	Number of brands	Minimum price INR (US$)	Maximum price INR (US$)	Cost ratio	Percentage cost variation
Bevacizumab (L01XC07)	Injection	100 mg	10	9,500 (114.85)	32,250 (389.87)	3.39	239.47
	Injection	400 mg	10	35,120 (424.56)	117,625 (1,421.97)	3.34	234.92
Trastuzumab (L01XC03)	Injection	150 mg	7	19,132.65 (231.29)	22,447 (271.36)	1.17	17.32
	Injection	440 mg	11	19,995 (241.72)	75,000 (906.67)	3.75	275.09
Rituximab (L01XC02)	Injection	100 mg	12	5,299 (64.06)	16,000 (193.42)	3.01	201.94
	Injection	500 mg	16	30,285.60 (366.12)	80,000 (967.12)	2.64	164.15

Table 5 shows the cost variation in the class “tumor necrosis factor alpha” (TNFα). Here it can be observed that the maximum variation was shown by 40 mg of adalimumab, which was 55.55%; the minimum variation was also shown by 100 mg of infliximab, which was 28.24%.

**Table 5:** The cost variation among the drugs belonging to the TNFα class. mg: Milligram, IU: International unit; INR: Indian national rupees; US$: United States Dollar; 1.00 US$ = 82.72 INR. Source: reference 16.

Drug (ATC code)	Dosage form	Dose	Number of brands	Minimum price INR (US$)	Maximum price INR (US$)	Cost ratio	Percentage cost variation
Infiliximab (L04AB02)	Injection	100 mg	2	32,000 (386.85)	41,039 (496.12)	1.28	28.24
Adalimumab (L04AB04)	Injection	40 mg	7	16,071.40 (194.29)	25,000 (302.22)	1.55	55.55

Table 6 shows the cost variation in the class of “enzymes.” Here, the maximum variation was shown by urokinase 500,000 IU, which was at 108.28%, and the minimum variation was also shown by urokinase 750,000 IU, which was at 14.12%.

**Table 6:** The cost variation among the drugs belonging to the class of “enzymes.” IU: International unit; INR: Indian national rupees; US$: United States Dollar; 1.00 US$ = 82.72 INR. Source: [16].

Drug (ATC code)	Dosage form	Dose	Number of brands	Minimum price INR (US$)	Maximum price INR (US$)	Cost ratio	Percentage cost variation
Urokinase (B01AD04)	Injection	250,000 IU	8	1,190.47 (14.39)	2,290 (27.68)	1.92	92.36
	Injection	500,000 IU	11	1,752.38 (21.18)	3,650 (44.12)	2.08	108.28
	Injection	750,000 IU	5	4,300 (51.98)	4,907.40 (59.32)	1.14	14.12
Streptokinase (B01AD01)	Injection	750,000 IU	16	1,141 (13.79)	2,178.32 (26.33)	1.9	90.91
	Injection	1,500,000 IU	9	1,880.06 (22.72)	3,898.09 (47.12)	2.07	107.33

Out of the 23 different formulations analyzed, 10 formulations showed a cost variation greater than 100% in the maximum and minimum costs. Five formulations displayed a cost variation greater than 200%. Overall, the biologic pegfilgrastim 6 mg showed a maximum cost variation of 1,022.92%, and the minimum cost variation was shown by darbepoetin alfa 200 mcg, which was 13.07% (Tables 2-3).

Correlation analysis

**Figure 1:**
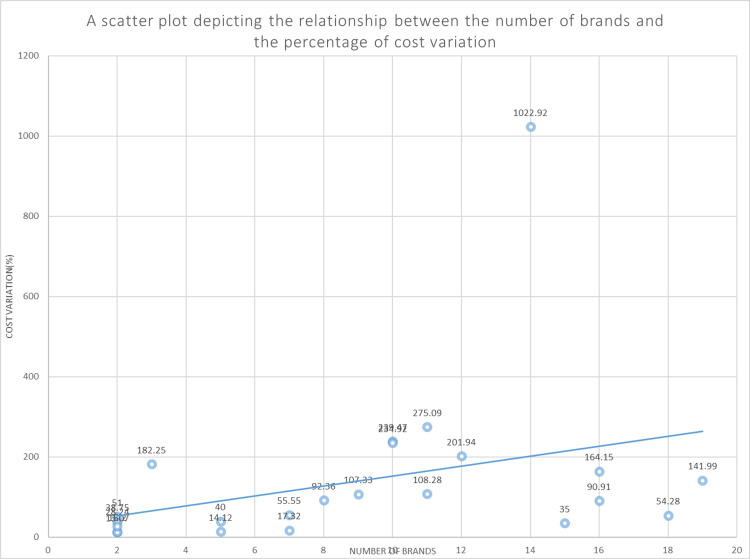
An impact of the number of brands available on cost variation. Correlation coefficient (r)-0.3305

A low correlation coefficient (r = 0.3305, 95% CI: -0.0945 to 0.6537, p = 0.1234) was obtained upon comparing the number of brands with cost variation. A low degree of association was established with the help of the “r” value of 0.3305 between the two variables (Figure 1).

## Discussion

Having utilized various search engines, few studies were found to have assessed the price variability of the various brands of biological drugs in India. Our findings discovered that prices of various biologic drugs manufactured by different brands in India show a wide variation in the minimum and maximum prices. In our study, however, rituximab 100 mg showed a cost variation of 201.94%, but the study by Adwal SK et al. (2018) [15] showed a cost variation of 40%. This was likely due to the changes in the prices set by the manufacturers. Furthermore, our findings revealed that the cost variation of urokinase 25,000 IU and 50,000 IU was at 92.36% and 108.28%, respectively. These results were substantiated by the study by Ray A et al. [14], which found a cost variation of 92.44% and 116.84%, respectively. In our study, the cost variation of streptokinase 75,000 IU was 90.12%, and the study by Ray A et al. [14], on the other hand, showed 84.34%.

Moreover, in our study, the cost variation of streptokinase 1,500,000 IU was 107.33%, while the study by Ray A et al. [14] showed 132.02%. Once the cost ratio exceeds two and the cost variation goes above 100%, the cost deviation is significant [17]. Out of the 23 different formulations analyzed, 10 formulations showed a variation greater than 100% in the maximum and minimum prices. Hence, there was a significant cost deviation in the 10 formulations of these biological drugs. The breakdown of immunologic tolerance is associated with the immune response against self-molecules. This results in autoimmune diseases [18]. In the majority of the cases, the cause of this is unknown. However, many studies that were carried out on this subject suggest associating environmental and genetic factors with certain types of infections [18]. According to population-based studies, autoimmune disorders (ADs) are the tenth leading cause of death in developing countries [19].

Currently, the mainstay of treatment in autoimmune diseases consists of disease-modifying anti-rheumatic drugs (DMARDs). However, when DMARDs cannot control a disease, biologic drugs have to be used as an adjunct to the above treatment [20]. They are also used in conditions like growth failure, growth hormone deficiency, anemia with chronic kidney disease, chemotherapy-induced anemia, chemotherapy-induced neutropenia, and various cancers. These diseases often require long-term treatment. Biologics have good efficacy and safety profiles, resulting in the rise of their utilization. This increased usage has also resulted from a better understanding of the primary targets of altered immune regulation and activity in diverse diseases [20]. These drugs, which have a targeted approach, are well tolerated by patients [20]. Although biologics have a high cost, this limits the number of patients who can afford biologics, ultimately putting a heavy financial burden on the healthcare system [8]. A large variation in the prices of the various brands of biologic medicines further complicates this situation. The availability of a wide range of biologics is beneficial to people who have severe or resistant diseases [20]. Severe diseases may be characterized by the involvement of vital organs that are disease-resistant or non-responsive to the conventional treatment modality, even biologic treatment [20]. Various drugs may augment treatment options from one class. If the usage of one TNFα blocker results in an adverse event, it can be changed to another TNFα blocker or an alternative but suitable class of medicines [20]. Even if a patient is not responding to a certain biologic over time, changing the biologic drug may result in a positive response. Better accessibility of various biologics will result in personalized treatment [20]. Hence, the availability of different biologic drugs will be advantageous to the patient; however, the high prices of biologics may cause a problem. Different brands of the same medicine differ in price from 2- to 100-fold [13]. There are many reasons for this cost variation; an example is that most drugs are under patent protection. Today’s market for new drugs is monopolistic in nature [13,21]. Retailers retain major influence over the cost of the product in the present market structure [13,21].

Biosimilar drug is a good option in place of costly and branded biologics. However, biosimilars are not the same as branded biologics. Biosimilars must undergo affixed tests to be recognized as interchangeable with biologics [8,22]. A biosimilar can be considered interchangeable with a biologic if it has equal clinical efficacy with the latter; there must also be no changes in safety when comparing their reference molecule [8,22]. If a biosimilar drug has been accepted as interchangeable, it may be used in place of the reference biologic without requiring a doctor’s consultation [8,22]. The government should encourage the production of biosimilars after the expiry of biologics patents. This makes biologic drugs affordable to the common man. Furthermore, various studies that compare biosimilar drugs with biologics must be encouraged. If possible, the approval process of biosimilar drugs should also be expedited [8]. India exports medicines to various countries at lower costs. Ironically, the country faces problems such as affordability and quality of medicine for its own people [9]. The main factor influencing this affordability is the fact that 70%-80% of active pharmaceutical ingredients are imported [23]. The manufacturing processes are exclusive and closely held secrets [24]. Many companies sell a certain medicine in the guise of various brand names other than the innovating firm [9]. This scenario has resulted in a wide variation of cost among the marketed medicines [9]. This issue has had serious economic consequences in India [9]. Every year, 63 million people in emergent nations like India are thrown into poverty due to unsustainable health spending [25]. In contrast to developed countries where medical insurance is well placed, people in developing countries like India have to pay the price of drugs out of their pockets [9]. After food, medicines are the second largest household expenditure item due to their meager availability in the civic sector, further pushing up domestic out-of-pocket expenditure (OOP) spending [25]. Thus, if a patient is prescribed a costly medicine brand, he has to unnecessarily pay the extra cost [9]. Various factors have fueled the increase of the burden of chronic disease in the South Asian region, which include genetic susceptibility, increased life expectancy, urbanization, automation, poor health services, etc. [25]. As stated by Saqib A et al. (2018) [26], people from low- and middle-income countries (LMIC) find it financially demanding to buy biologics; hence, this kind of treatment may be out of the question [25].

Pharmaceutical industries have a motive to develop medicines for cancer and chronic diseases because these diseases have high prevalence and require long-term treatment [25]. Biotech drugs have revolutionized the treatment of many ailments, including autoimmune diseases and cancer, but these medications may require significant OOP expenditure [25]. Inadequate data on the comparative costs and quality of medicines make it troublesome for physicians to prescribe the most economical ones [25]. Thus regulators should ensure on allowing only quality products enter the market. Physicians may also be influenced by biased information, which is in the form of promotional literature, formularies, and marketing tactics of the medical representatives; this narrows the options for both the physician and patient [13,21]. This problem could be addressed by continuing medical education. The government of India issued a drug price control order (DPCO) in 2013 to regulate the prices of drugs. The national list of essential medicines in 2015 covered 376 medicines [13]. The medicines whose prices are governed by DPCO cannot be retailed at a cost greater than that fixed by the government [13,27]. However, there is a limited number of biologic medicines under the supervision of the DPCO. Hence, the prices of very few biologics can be fixed by the DPCO. More biologics should be brought under DPCO for patient affordability. Transatlantic Trade and Investment Partnership (TTIP) and Trans-Pacific Partnership (TPP) accords exemplify trade and investment agreements that may exert an effect on pharmaceutical prices and efficacy prices, such as prolonging patent terms, depressing patentability norms, market/data uniqueness averting generic and biosimilar drugs from entering the market, data safety obligations for biologics allowing high costs of cancer biologic drugs to remain on the market for longer, and ways to block generic medicines from entering the nation are all examples of far-reaching intellectual property standards backing the monopoly of large medicine manufacturers [24]. The TRIPS-Plus provisions in the treaties may disproportionately affect the emergent nations by allowing price increases for vital medications if they are properly translated into domestic law. Policymakers play a critical role in negotiating rules and laws [24]. Various policy changes or interventions, which can aid in decreasing the price of biologic medicine, are breaking out of manufacturers' control. Hence, governing guidelines for various administrative agencies should be formulated, favoring manufacturers with cheaper and cost-effective drugs. Additionally, the approval of newer biologics should be expedited. A balance should be achieved between the treating physician's autonomy in prescribing the biologic drugs and the cost suffered by the patients. Furthermore, tax incentives and other measures should be taken to encourage generic nonprofit companies [25].

Limitations

Studies like this should be conducted on a bigger scale on the same therapeutic class in order to get beyond the limitation that only a few brands of biologics from the CIMS website were taken into consideration.

## Conclusions

Our study revealed significant variances in the prices of various biologic drugs in India. This knowledge allows the physician to consider cost deviation as they prescribe different brands of drugs. This enables them to choose efficacious, cost-efficient drugs to benefit the patient. It is recommended that the government authorities consider policy changes such as controlling the monopolistic practices of manufacturers to promote the common good.
